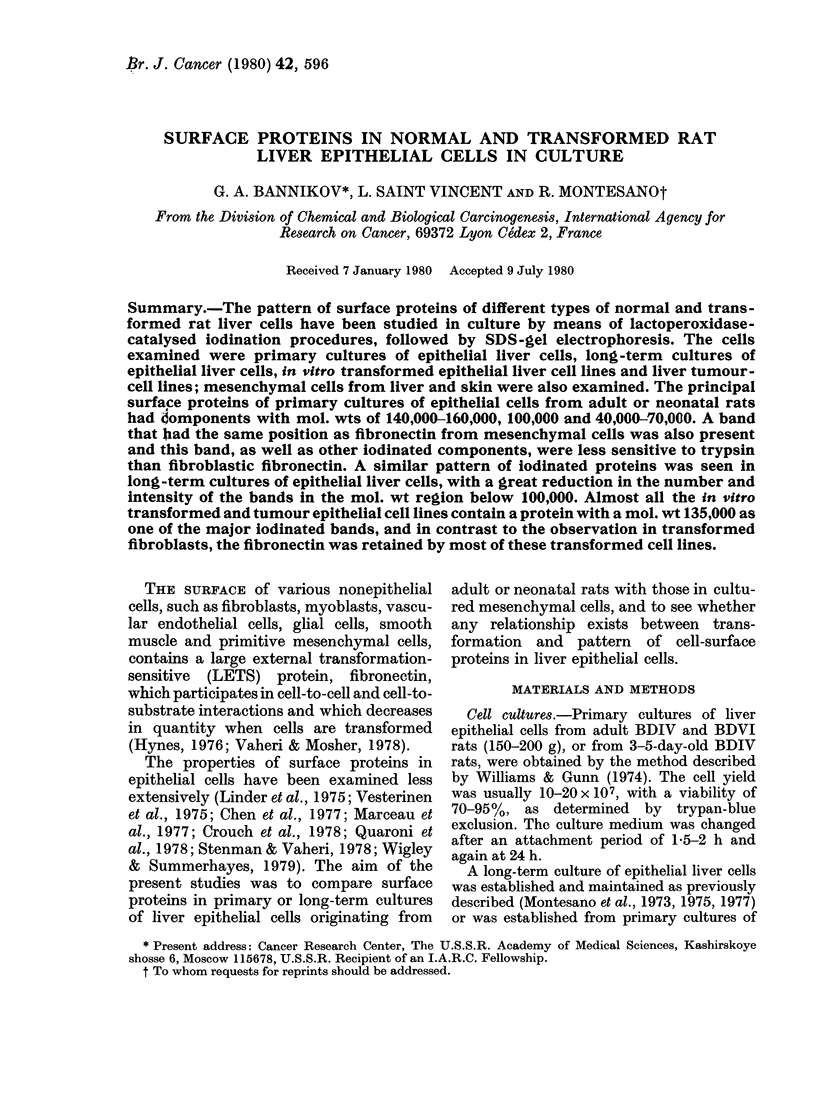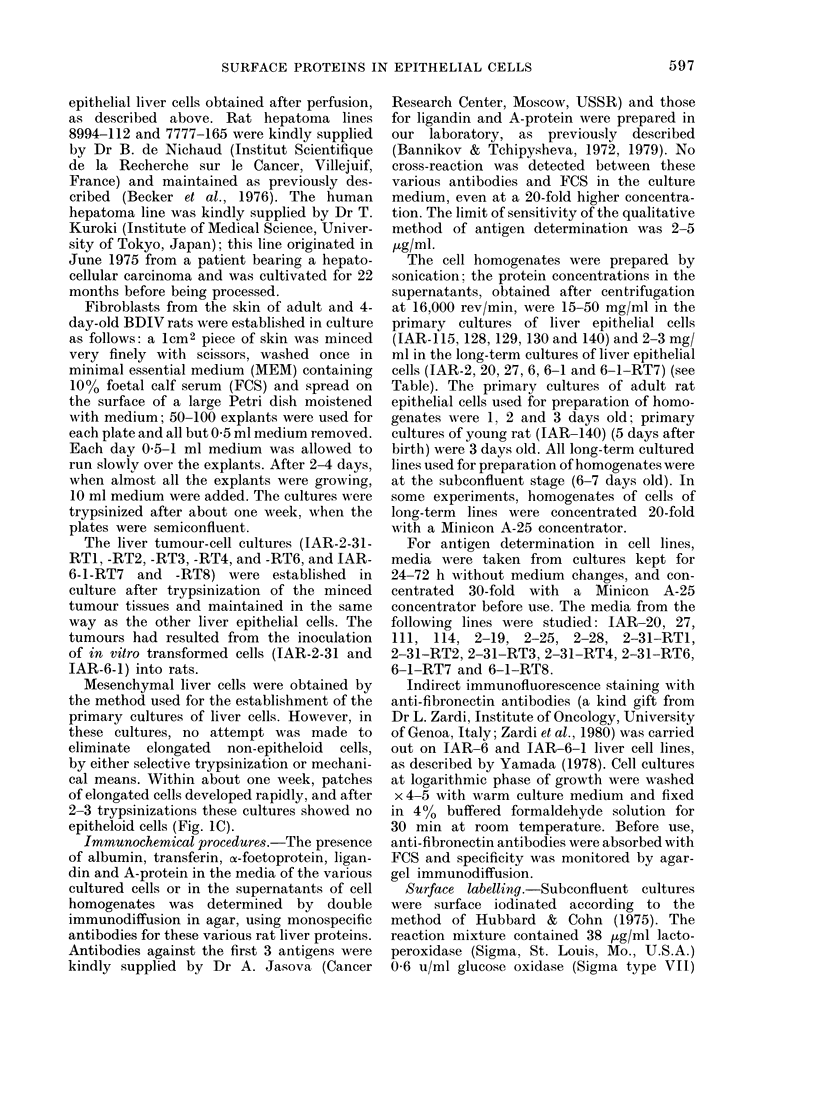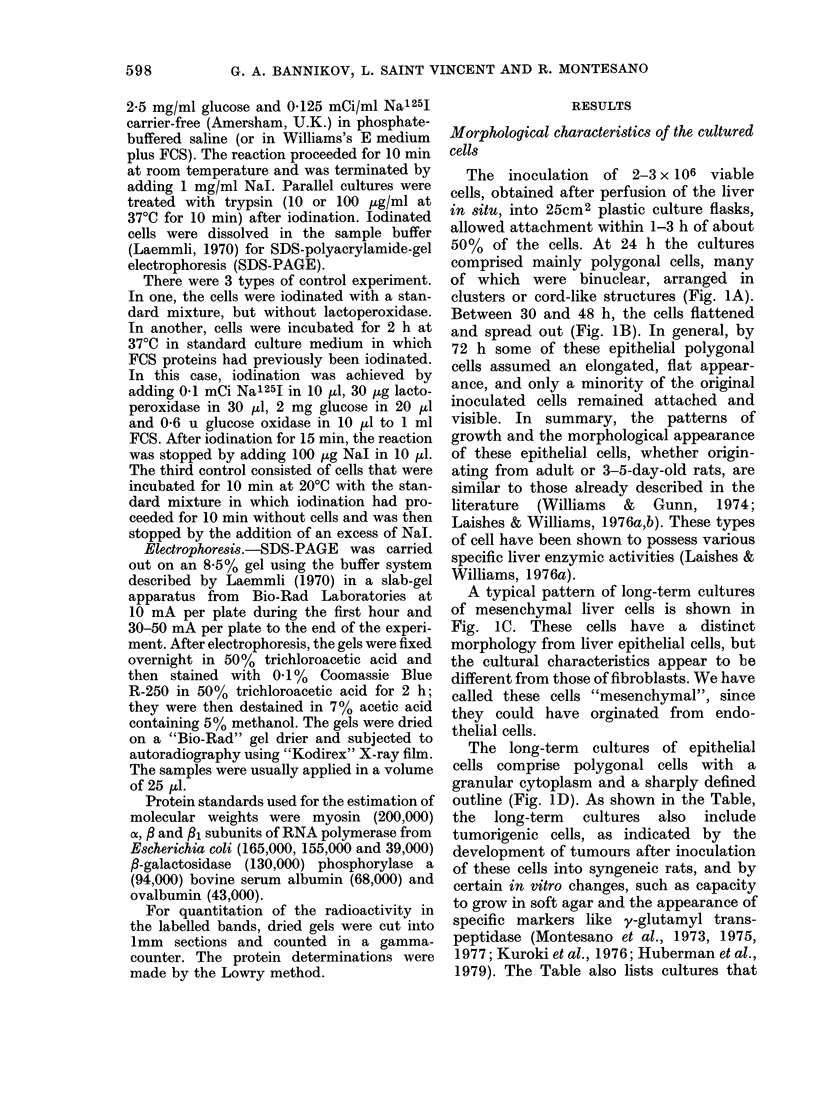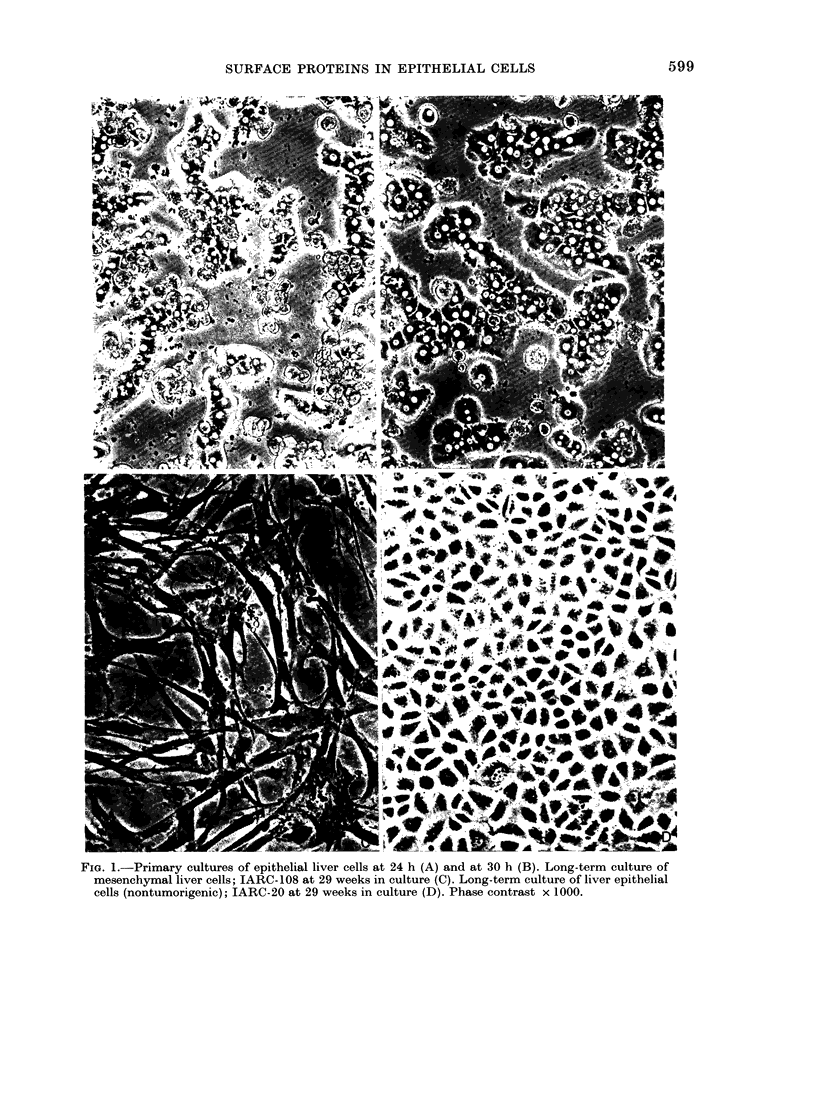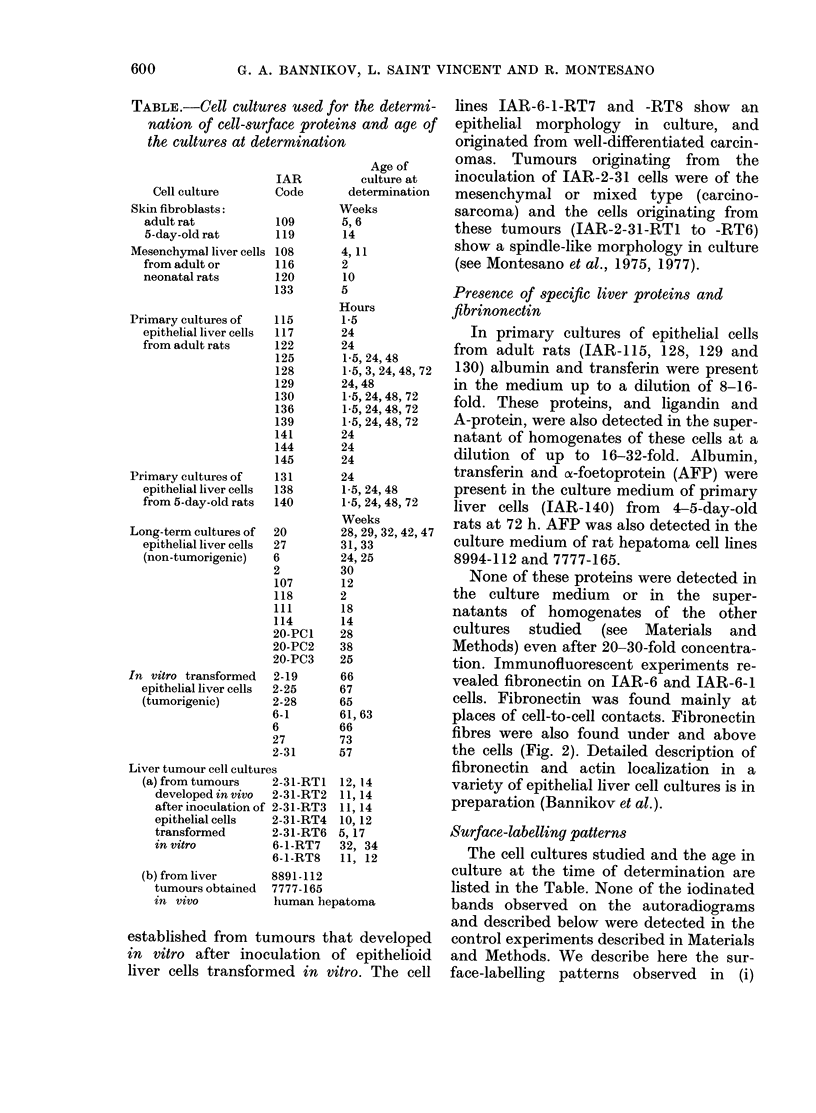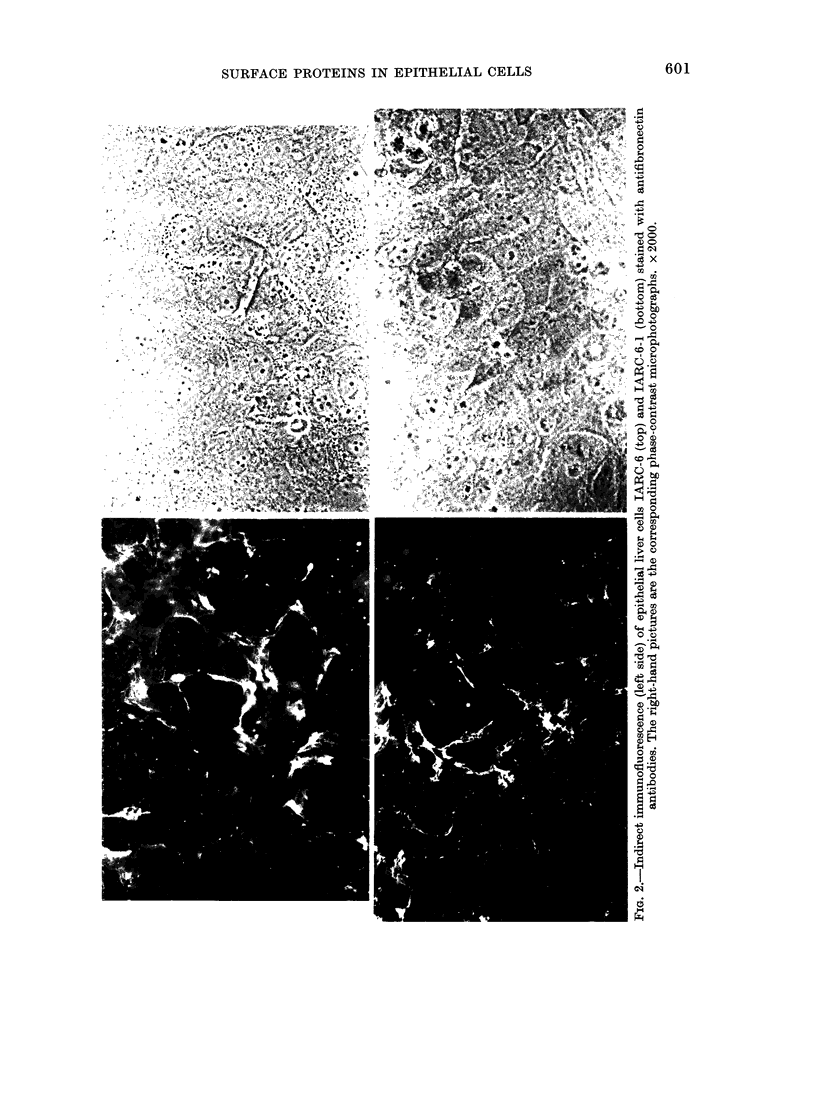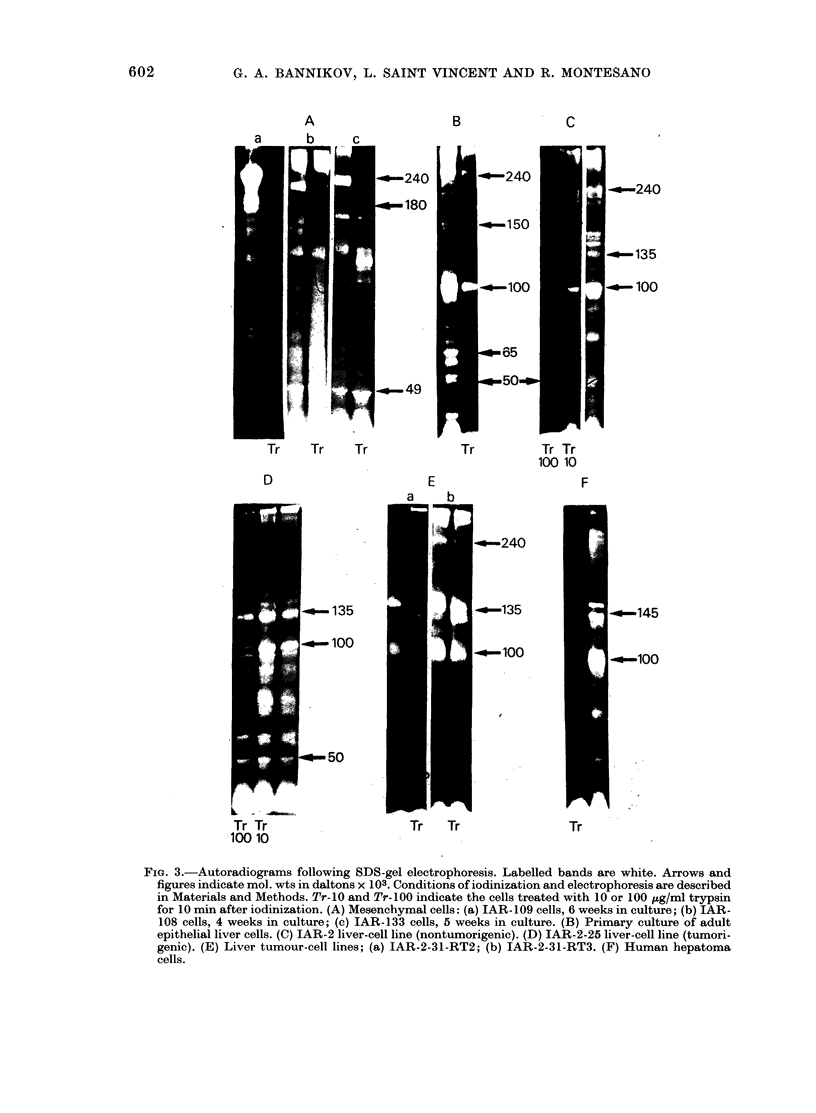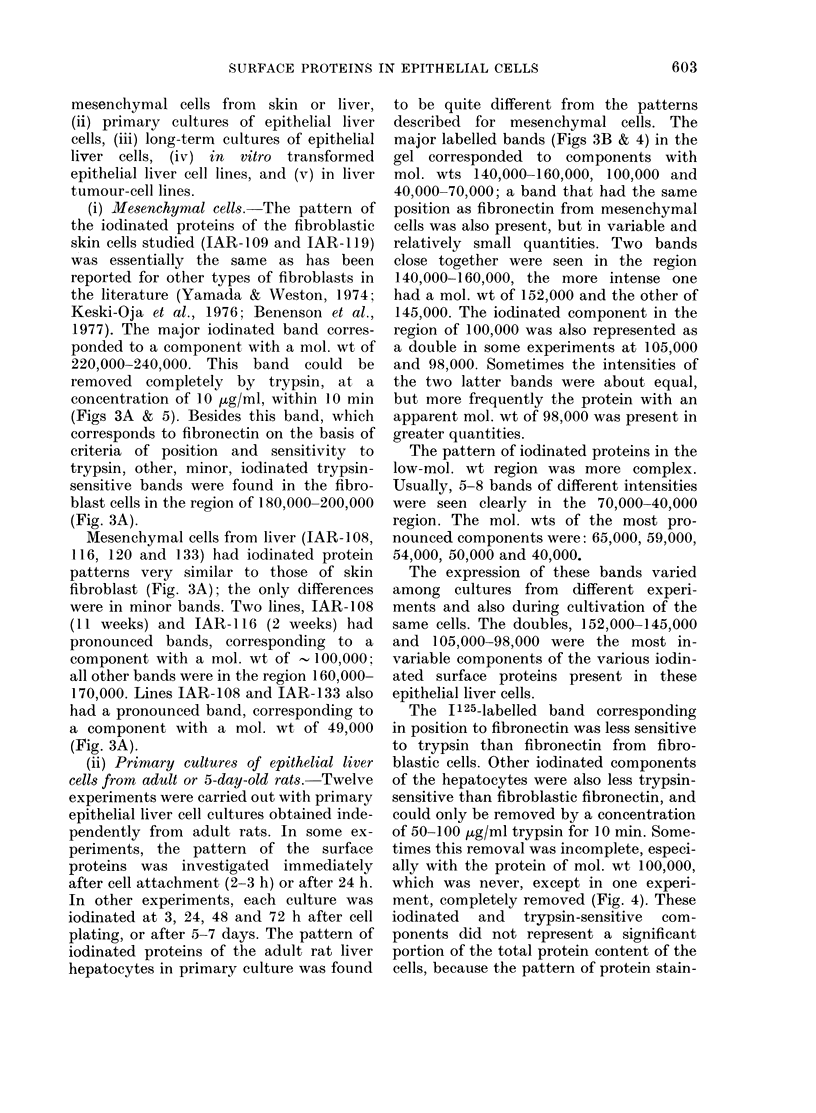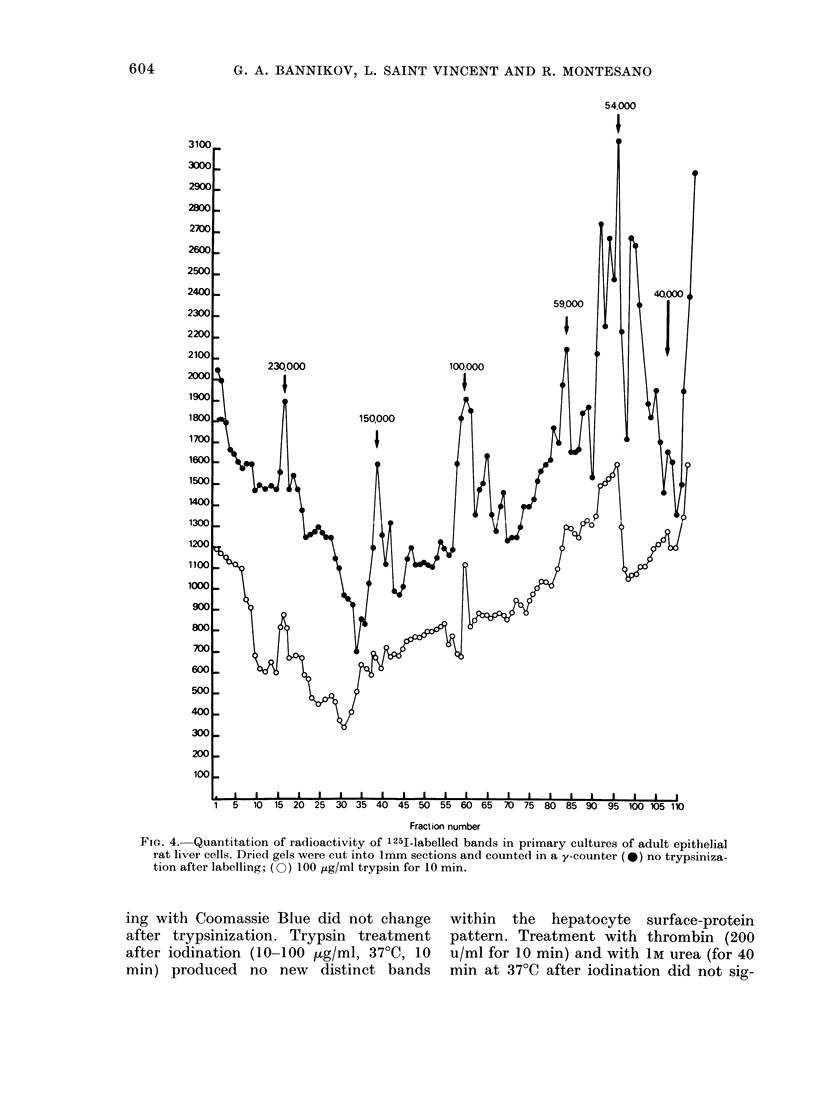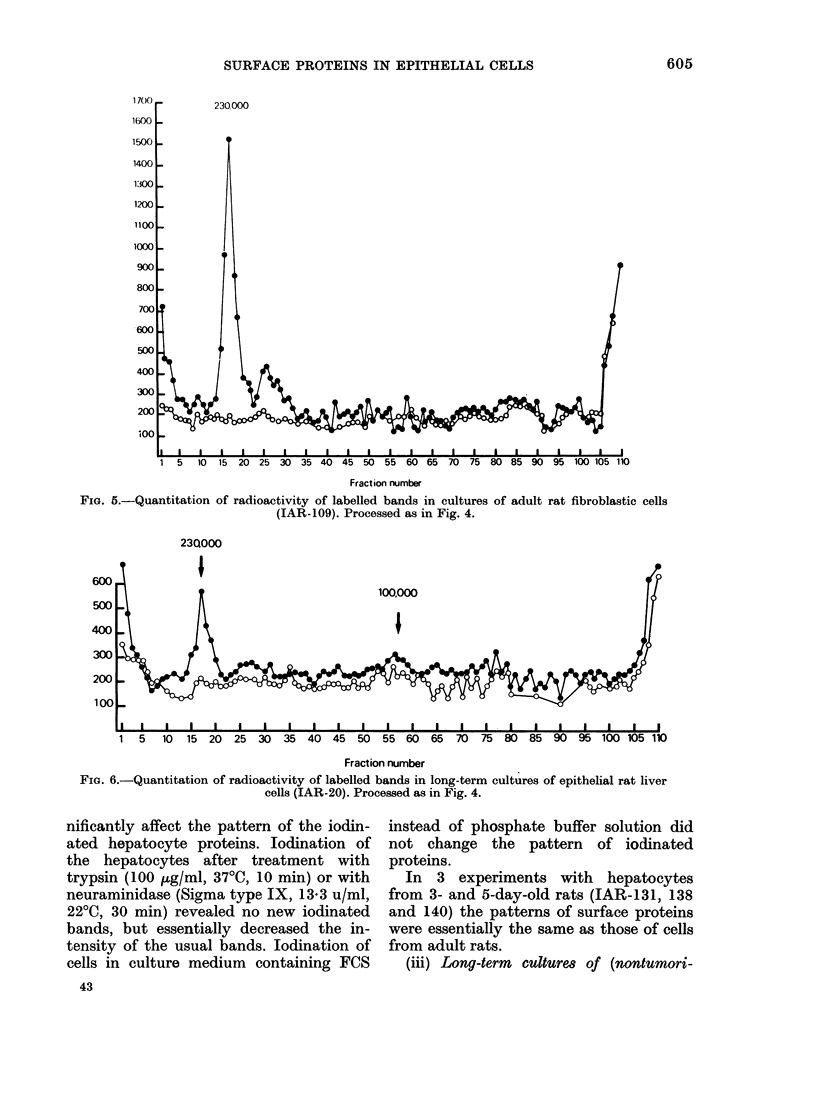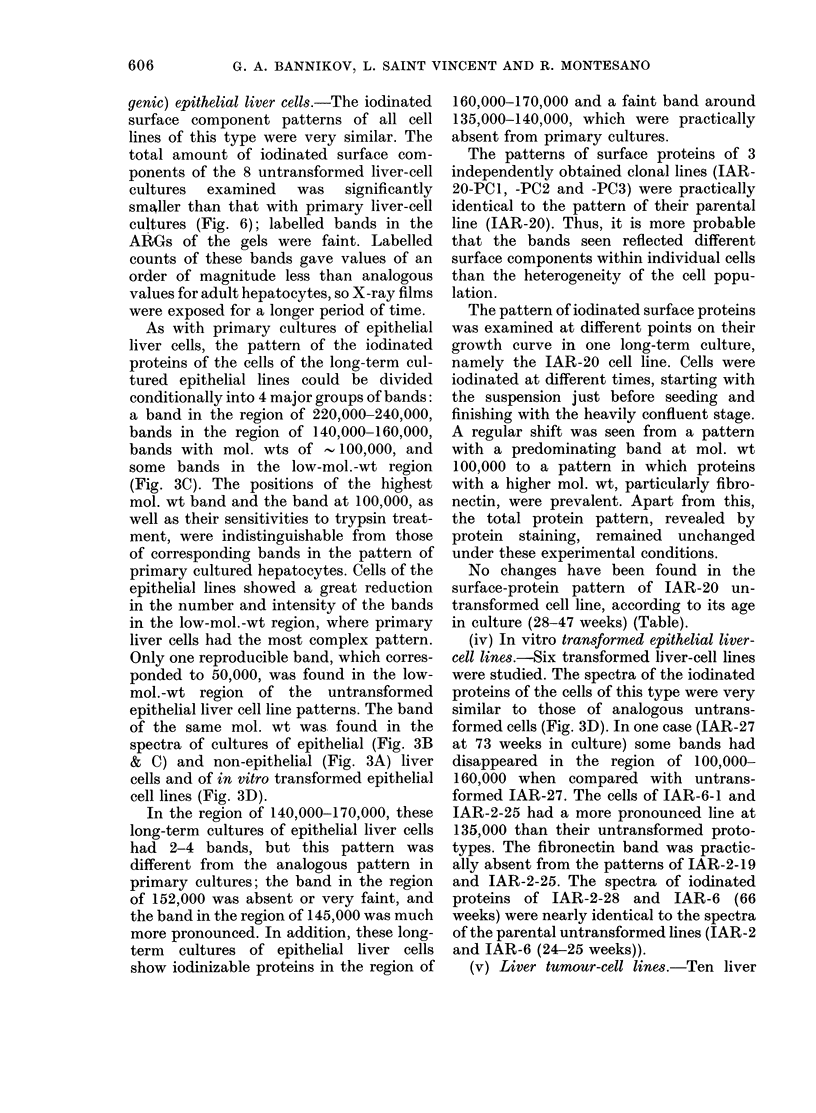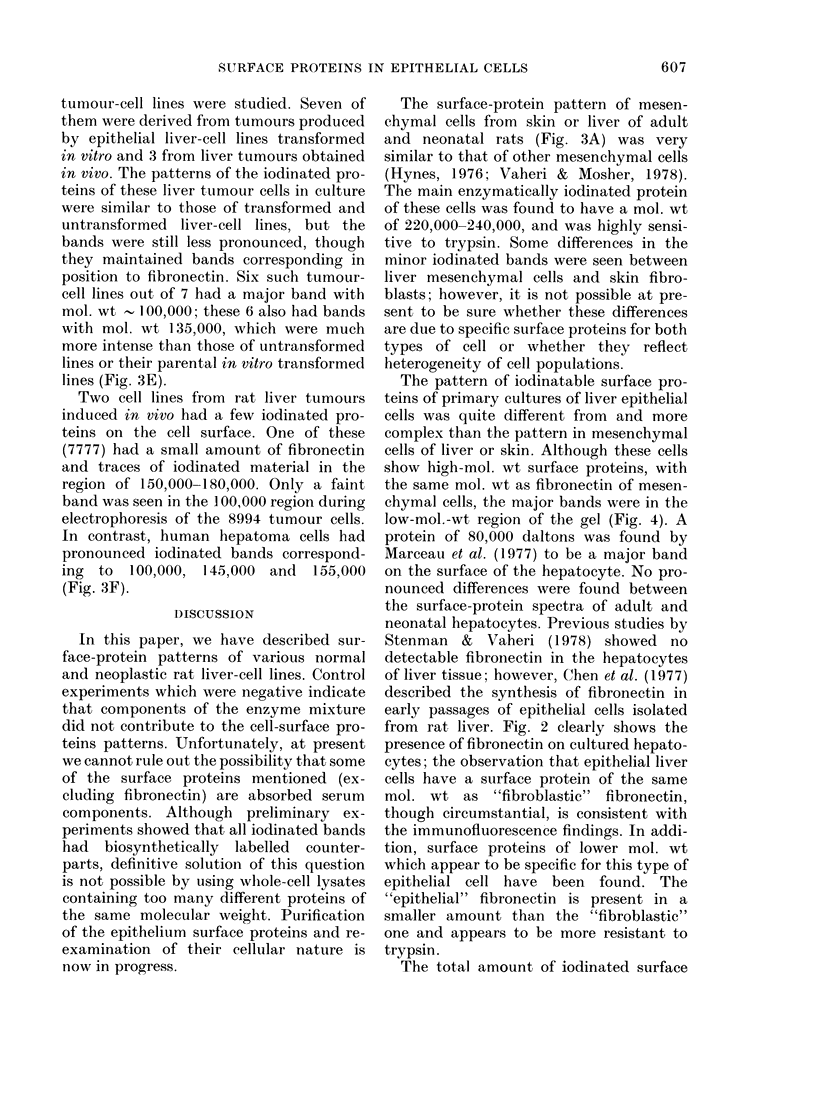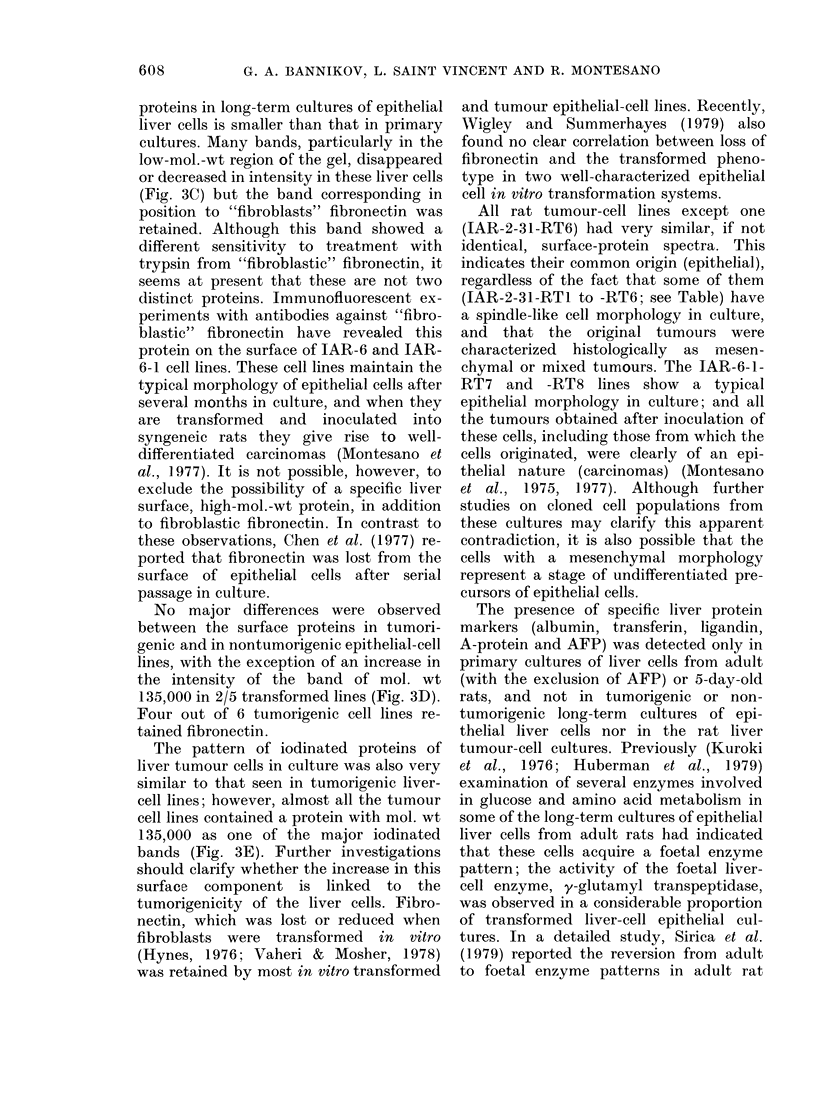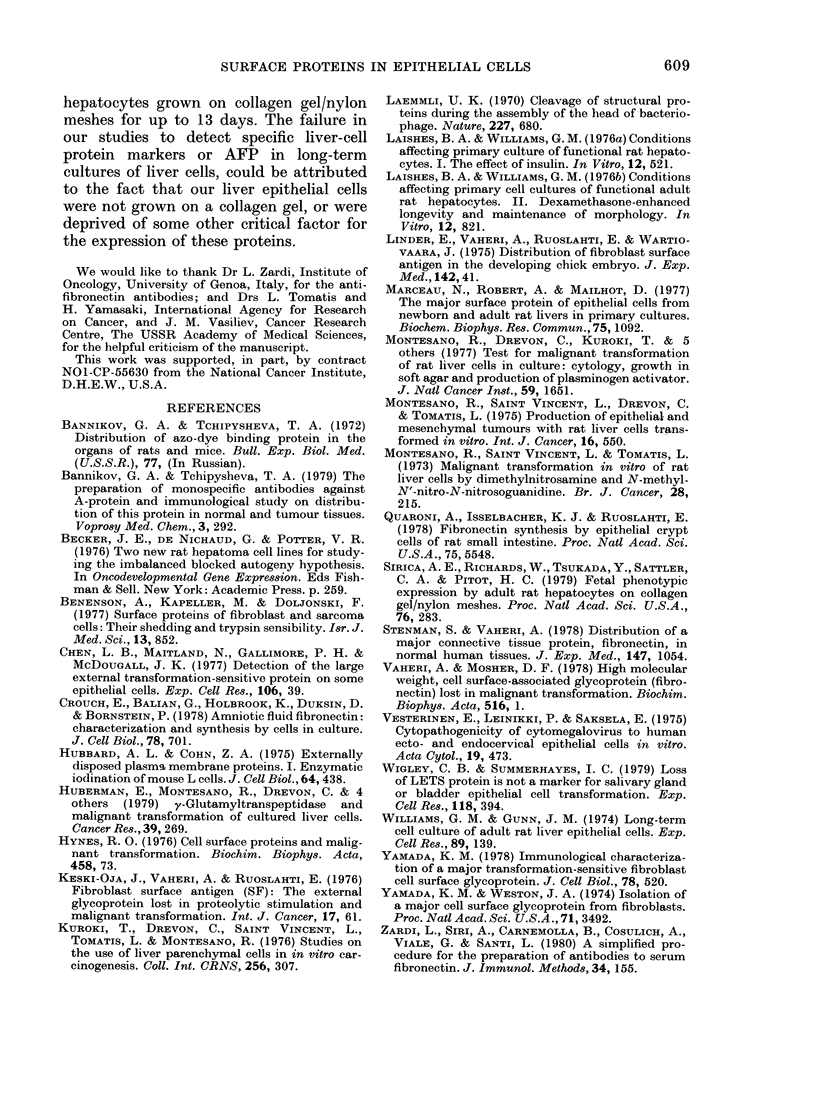# Surface proteins in normal and transformed rat liver epithelial cells in culture.

**DOI:** 10.1038/bjc.1980.284

**Published:** 1980-10

**Authors:** G. A. Bannikov, L. Saint Vincent, R. Montesano

## Abstract

**Images:**


Br. J. Cancer (1980) 42, 596

### SURFACE PROTEINS IN NORMAL AND TRANSFORMED RAT LIVER EPITHELIAL CELLS IN CULTURE

G. A. BANNIKOV*, L. SAINT VINCENT AND R. MONTESANO†

*From the Division of Chemical and Biological Carcinogenesis, International Agency for Research on Cancer, 69372 Lyon Cédex 2, France*

Received 7 January 1980 Accepted 9 July 1980

**Summary.—The pattern of surface proteins of different types of normal and transformed rat liver cells have been studied in culture by means of lactoperoxidase-catalysed iodination procedures, followed by SDS-gel electrophoresis. The cells examined were primary cultures of epithelial liver cells, long-term cultures of epithelial liver cells, *in vitro* transformed epithelial liver cell lines and liver tumour-cell lines; mesenchymal cells from liver and skin were also examined. The principal surface proteins of primary cultures of epithelial cells from adult or neonatal rats had components with mol. wts of 140,000–160,000, 100,000 and 40,000–70,000. A band that had the same position as fibronectin from mesenchymal cells was also present and this band, as well as other iodinated components, were less sensitive to trypsin than fibroblastic fibronectin. A similar pattern of iodinated proteins was seen in long-term cultures of epithelial liver cells, with a great reduction in the number and intensity of the bands in the mol. wt region below 100,000. Almost all the *in vitro* transformed and tumour epithelial cell lines contain a protein with a mol. wt 135,000 as one of the major iodinated bands, and in contrast to the observation in transformed fibroblasts, the fibronectin was retained by most of these transformed cell lines.**

THE SURFACE of various nonepithelial cells, such as fibroblasts, myoblasts, vascular endothelial cells, glial cells, smooth muscle and primitive mesenchymal cells, contains a large external transformation-sensitive (LETS) protein, fibronectin, which participates in cell-to-cell and cell-to-substrate interactions and which decreases in quantity when cells are transformed (Hynes, 1976; Vaheri & Mosher, 1978).

The properties of surface proteins in epithelial cells have been examined less extensively (Linder *et al.*, 1975; Vesterinen *et al.*, 1975; Chen *et al.*, 1977; Marceau *et al.*, 1977; Crouch *et al.*, 1978; Quaroni *et al.*, 1978; Stenman & Vaheri, 1978; Wigley & Summerhayes, 1979). The aim of the present studies was to compare surface proteins in primary or long-term cultures of liver epithelial cells originating from adult or neonatal rats with those in cultured mesenchymal cells, and to see whether any relationship exists between transformation and pattern of cell-surface proteins in liver epithelial cells.

### MATERIALS AND METHODS

*Cell cultures.*—Primary cultures of liver epithelial cells from adult BDIV and BDVI rats (150–200 g), or from 3–5-day-old BDIV rats, were obtained by the method described by Williams & Gunn (1974). The cell yield was usually 10–20 × $10^7$, with a viability of 70–95%, as determined by trypan-blue exclusion. The culture medium was changed after an attachment period of 1·5–2 h and again at 24 h.

A long-term culture of epithelial liver cells was established and maintained as previously described (Montesano *et al.*, 1973, 1975, 1977) or was established from primary cultures of

* Present address: Cancer Research Center, The U.S.S.R. Academy of Medical Sciences, Kashirskoye shosse 6, Moscow 115678, U.S.S.R. Recipient of an I.A.R.C. Fellowship.

† To whom requests for reprints should be addressed.

epithelial liver cells obtained after perfusion, as described above. Rat hepatoma lines 8994–112 and 7777–165 were kindly supplied by Dr B. de Nichaud (Institut Scientifique de la Recherche sur le Cancer, Villejuif, France) and maintained as previously described (Becker *et al.*, 1976). The human hepatoma line was kindly supplied by Dr T. Kuroki (Institute of Medical Science, University of Tokyo, Japan); this line originated in June 1975 from a patient bearing a hepatocellular carcinoma and was cultivated for 22 months before being processed.

Fibroblasts from the skin of adult and 4-day-old BDIV rats were established in culture as follows: a 1cm$^2$ piece of skin was minced very finely with scissors, washed once in minimal essential medium (MEM) containing 10% foetal calf serum (FCS) and spread on the surface of a large Petri dish moistened with medium; 50–100 explants were used for each plate and all but 0·5 ml medium removed. Each day 0·5–1 ml medium was allowed to run slowly over the explants. After 2–4 days, when almost all the explants were growing, 10 ml medium were added. The cultures were trypsinized after about one week, when the plates were semiconfluent.

The liver tumour-cell cultures (IAR-2-31-RT1, -RT2, -RT3, -RT4, and -RT6, and IAR-6-1-RT7 and -RT8) were established in culture after trypsinization of the minced tumour tissues and maintained in the same way as the other liver epithelial cells. The tumours had resulted from the inoculation of *in vitro* transformed cells (IAR-2-31 and IAR-6-1) into rats.

Mesenchymal liver cells were obtained by the method used for the establishment of the primary cultures of liver cells. However, in these cultures, no attempt was made to eliminate elongated non-epitheloid cells, by either selective trypsinization or mechanical means. Within about one week, patches of elongated cells developed rapidly, and after 2–3 trypsinizations these cultures showed no epitheloid cells (Fig. 1C).

*Immunochemical procedures.*—The presence of albumin, transferin, α-foetoprotein, ligandin and A-protein in the media of the various cultured cells or in the supernatants of cell homogenates was determined by double immunodiffusion in agar, using monospecific antibodies for these various rat liver proteins. Antibodies against the first 3 antigens were kindly supplied by Dr A. Jasova (Cancer Research Center, Moscow, USSR) and those for ligandin and A-protein were prepared in our laboratory, as previously described (Bannikov & Tchipysheva, 1972, 1979). No cross-reaction was detected between these various antibodies and FCS in the culture medium, even at a 20-fold higher concentration. The limit of sensitivity of the qualitative method of antigen determination was 2–5 μg/ml.

The cell homogenates were prepared by sonication; the protein concentrations in the supernatants, obtained after centrifugation at 16,000 rev/min, were 15–50 mg/ml in the primary cultures of liver epithelial cells (IAR-115, 128, 129, 130 and 140) and 2–3 mg/ml in the long-term cultures of liver epithelial cells (IAR-2, 20, 27, 6, 6–1 and 6–1–RT7) (see Table). The primary cultures of adult rat epithelial cells used for preparation of homogenates were 1, 2 and 3 days old; primary cultures of young rat (IAR–140) (5 days after birth) were 3 days old. All long-term cultured lines used for preparation of homogenates were at the subconfluent stage (6–7 days old). In some experiments, homogenates of cells of long-term lines were concentrated 20-fold with a Minicon A-25 concentrator.

For antigen determination in cell lines, media were taken from cultures kept for 24–72 h without medium changes, and concentrated 30-fold with a Minicon A-25 concentrator before use. The media from the following lines were studied: IAR–20, 27, 111, 114, 2–19, 2–25, 2–28, 2–31–RT1, 2–31–RT2, 2–31–RT3, 2–31–RT4, 2–31–RT6, 6–1–RT7 and 6–1–RT8.

Indirect immunofluorescence staining with anti-fibronectin antibodies (a kind gift from Dr L. Zardi, Institute of Oncology, University of Genoa, Italy; Zardi *et al.*, 1980) was carried out on IAR–6 and IAR–6–1 liver cell lines, as described by Yamada (1978). Cell cultures at logarithmic phase of growth were washed ×4–5 with warm culture medium and fixed in 4% buffered formaldehyde solution for 30 min at room temperature. Before use, anti-fibronectin antibodies were absorbed with FCS and specificity was monitored by agar-gel immunodiffusion.

*Surface labelling.*—Subconfluent cultures were surface iodinated according to the method of Hubbard & Cohn (1975). The reaction mixture contained 38 μg/ml lactoperoxidase (Sigma, St. Louis, Mo., U.S.A.) 0·6 u/ml glucose oxidase (Sigma type VII)

2·5 mg/ml glucose and 0·125 mCi/ml $Na^{125}I$ carrier-free (Amersham, U.K.) in phosphate-buffered saline (or in Williams's E medium plus FCS). The reaction proceeded for 10 min at room temperature and was terminated by adding 1 mg/ml NaI. Parallel cultures were treated with trypsin (10 or 100 μg/ml at 37°C for 10 min) after iodination. Iodinated cells were dissolved in the sample buffer (Laemmli, 1970) for SDS-polyacrylamide-gel electrophoresis (SDS-PAGE).

There were 3 types of control experiment. In one, the cells were iodinated with a standard mixture, but without lactoperoxidase. In another, cells were incubated for 2 h at 37°C in standard culture medium in which FCS proteins had previously been iodinated. In this case, iodination was achieved by adding 0·1 mCi $Na^{125}I$ in 10 μl, 30 μg lactoperoxidase in 30 μl, 2 mg glucose in 20 μl and 0·6 u glucose oxidase in 10 μl to 1 ml FCS. After iodination for 15 min, the reaction was stopped by adding 100 μg NaI in 10 μl. The third control consisted of cells that were incubated for 10 min at 20°C with the standard mixture in which iodination had proceeded for 10 min without cells and was then stopped by the addition of an excess of NaI.

*Electrophoresis.*—SDS-PAGE was carried out on an 8·5% gel using the buffer system described by Laemmli (1970) in a slab-gel apparatus from Bio-Rad Laboratories at 10 mA per plate during the first hour and 30–50 mA per plate to the end of the experiment. After electrophoresis, the gels were fixed overnight in 50% trichloroacetic acid and then stained with 0·1% Coomassie Blue R-250 in 50% trichloroacetic acid for 2 h; they were then destained in 7% acetic acid containing 5% methanol. The gels were dried on a "Bio-Rad" gel drier and subjected to autoradiography using "Kodirex" X-ray film. The samples were usually applied in a volume of 25 μl.

Protein standards used for the estimation of molecular weights were myosin (200,000) α, β and $\beta_1$ subunits of RNA polymerase from *Escherichia coli* (165,000, 155,000 and 39,000) β-galactosidase (130,000) phosphorylase a (94,000) bovine serum albumin (68,000) and ovalbumin (43,000).

For quantitation of the radioactivity in the labelled bands, dried gels were cut into 1mm sections and counted in a gamma-counter. The protein determinations were made by the Lowry method.

### RESULTS

### *Morphological characteristics of the cultured cells*

The inoculation of $2\text{–}3 \times 10^6$ viable cells, obtained after perfusion of the liver *in situ*, into 25cm² plastic culture flasks, allowed attachment within 1–3 h of about 50% of the cells. At 24 h the cultures comprised mainly polygonal cells, many of which were binuclear, arranged in clusters or cord-like structures (Fig. 1A). Between 30 and 48 h, the cells flattened and spread out (Fig. 1B). In general, by 72 h some of these epithelial polygonal cells assumed an elongated, flat appearance, and only a minority of the original inoculated cells remained attached and visible. In summary, the patterns of growth and the morphological appearance of these epithelial cells, whether originating from adult or 3–5-day-old rats, are similar to those already described in the literature (Williams & Gunn, 1974; Laishes & Williams, 1976*a*,*b*). These types of cell have been shown to possess various specific liver enzymic activities (Laishes & Williams, 1976*a*).

A typical pattern of long-term cultures of mesenchymal liver cells is shown in Fig. 1C. These cells have a distinct morphology from liver epithelial cells, but the cultural characteristics appear to be different from those of fibroblasts. We have called these cells "mesenchymal", since they could have orginated from endothelial cells.

The long-term cultures of epithelial cells comprise polygonal cells with a granular cytoplasm and a sharply defined outline (Fig. 1D). As shown in the Table, the long-term cultures also include tumorigenic cells, as indicated by the development of tumours after inoculation of these cells into syngeneic rats, and by certain *in vitro* changes, such as capacity to grow in soft agar and the appearance of specific markers like γ-glutamyl transpeptidase (Montesano *et al.*, 1973, 1975, 1977; Kuroki *et al.*, 1976; Huberman *et al.*, 1979). The Table also lists cultures that

FIG. 1.—Primary cultures of epithelial liver cells at 24 h (A) and at 30 h (B). Long-term culture of mesenchymal liver cells; IARC-108 at 29 weeks in culture (C). Long-term culture of liver epithelial cells (nontumorigenic); IARC-20 at 29 weeks in culture (D). Phase contrast ×1000.

TABLE.—*Cell cultures used for the determination of cell-surface proteins and age of the cultures at determination*

| Cell culture | IAR Code | Age of culture at determination |
|---|---|---|
| Skin fibroblasts: | | Weeks |
| adult rat | 109 | 5, 6 |
| 5-day-old rat | 119 | 14 |
| Mesenchymal liver cells | 108 | 4, 11 |
| from adult or | 116 | 2 |
| neonatal rats | 120 | 10 |
| | 133 | 5 |
| | | Hours |
| Primary cultures of | 115 | 1·5 |
| epithelial liver cells | 117 | 24 |
| from adult rats | 122 | 24 |
| | 125 | 1·5, 24, 48 |
| | 128 | 1·5, 3, 24, 48, 72 |
| | 129 | 24, 48 |
| | 130 | 1·5, 24, 48, 72 |
| | 136 | 1·5, 24, 48, 72 |
| | 139 | 1·5, 24, 48, 72 |
| | 141 | 24 |
| | 144 | 24 |
| | 145 | 24 |
| Primary cultures of | 131 | 24 |
| epithelial liver cells | 138 | 1·5, 24, 48 |
| from 5-day-old rats | 140 | 1·5, 24, 48, 72 |
| | | Weeks |
| Long-term cultures of | 20 | 28, 29, 32, 42, 47 |
| epithelial liver cells | 27 | 31, 33 |
| (non-tumorigenic) | 6 | 24, 25 |
| | 2 | 30 |
| | 107 | 12 |
| | 118 | 2 |
| | 111 | 18 |
| | 114 | 14 |
| | 20-PC1 | 28 |
| | 20-PC2 | 38 |
| | 20-PC3 | 25 |
| *In vitro* transformed | 2-19 | 66 |
| epithelial liver cells | 2-25 | 67 |
| (tumorigenic) | 2-28 | 65 |
| | 6-1 | 61, 63 |
| | 6 | 66 |
| | 27 | 73 |
| | 2-31 | 57 |
| Liver tumour cell cultures | | |
| (a) from tumours | 2-31-RT1 | 12, 14 |
| developed *in vivo* | 2-31-RT2 | 11, 14 |
| after inoculation of | 2-31-RT3 | 11, 14 |
| epithelial cells | 2-31-RT4 | 10, 12 |
| transformed | 2-31-RT6 | 5, 17 |
| *in vitro* | 6-1-RT7 | 32, 34 |
| | 6-1-RT8 | 11, 12 |
| (b) from liver | 8891-112 | |
| tumours obtained | 7777-165 | |
| *in vivo* | human hepatoma | |

established from tumours that developed *in vitro* after inoculation of epithelioid liver cells transformed *in vitro*. The cell lines IAR-6-1-RT7 and -RT8 show an epithelial morphology in culture, and originated from well-differentiated carcinomas. Tumours originating from the inoculation of IAR-2-31 cells were of the mesenchymal or mixed type (carcinosarcoma) and the cells originating from these tumours (IAR-2-31-RT1 to -RT6) show a spindle-like morphology in culture (see Montesano *et al.*, 1975, 1977).

*Presence of specific liver proteins and fibrinonectin*

In primary cultures of epithelial cells from adult rats (IAR-115, 128, 129 and 130) albumin and transferin were present in the medium up to a dilution of 8–16-fold. These proteins, and ligandin and A-protein, were also detected in the supernatant of homogenates of these cells at a dilution of up to 16–32-fold. Albumin, transferin and $\alpha$-foetoprotein (AFP) were present in the culture medium of primary liver cells (IAR-140) from 4–5-day-old rats at 72 h. AFP was also detected in the culture medium of rat hepatoma cell lines 8994-112 and 7777-165.

None of these proteins were detected in the culture medium or in the supernatants of homogenates of the other cultures studied (see Materials and Methods) even after 20–30-fold concentration. Immunofluorescent experiments revealed fibronectin on IAR-6 and IAR-6-1 cells. Fibronectin was found mainly at places of cell-to-cell contacts. Fibronectin fibres were also found under and above the cells (Fig. 2). Detailed description of fibronectin and actin localization in a variety of epithelial liver cell cultures is in preparation (Bannikov *et al.*).

*Surface-labelling patterns*

The cell cultures studied and the age in culture at the time of determination are listed in the Table. None of the iodinated bands observed on the autoradiograms and described below were detected in the control experiments described in Materials and Methods. We describe here the surface-labelling patterns observed in (i)

FIG. 2.—Indirect immunofluorescence (left side) of epithelial liver cells IARC-6 (top) and IARC-6-1 (bottom) stained with antifibronectin antibodies. The right-hand pictures are the corresponding phase-contrast microphotographs. × 2000.

FIG. 3.—Autoradiograms following SDS-gel electrophoresis. Labelled bands are white. Arrows and figures indicate mol. wts in daltons $\times 10^3$. Conditions of iodinization and electrophoresis are described in Materials and Methods. *Tr*-10 and *Tr*-100 indicate the cells treated with 10 or 100 $\mu$g/ml trypsin for 10 min after iodinization. (A) Mesenchymal cells: (a) IAR-109 cells, 6 weeks in culture; (b) IAR-108 cells, 4 weeks in culture; (c) IAR-133 cells, 5 weeks in culture. (B) Primary culture of adult epithelial liver cells. (C) IAR-2 liver-cell line (nontumorigenic). (D) IAR-2-25 liver-cell line (tumorigenic). (E) Liver tumour-cell lines; (a) IAR-2-31-RT2; (b) IAR-2-31-RT3. (F) Human hepatoma cells.

mesenchymal cells from skin or liver, (ii) primary cultures of epithelial liver cells, (iii) long-term cultures of epithelial liver cells, (iv) *in vitro* transformed epithelial liver cell lines, and (v) in liver tumour-cell lines.

(i) *Mesenchymal cells.*—The pattern of the iodinated proteins of the fibroblastic skin cells studied (IAR-109 and IAR-119) was essentially the same as has been reported for other types of fibroblasts in the literature (Yamada & Weston, 1974; Keski-Oja *et al.*, 1976; Benenson *et al.*, 1977). The major iodinated band corresponded to a component with a mol. wt of 220,000–240,000. This band could be removed completely by trypsin, at a concentration of 10 $\mu$g/ml, within 10 min (Figs 3A & 5). Besides this band, which corresponds to fibronectin on the basis of criteria of position and sensitivity to trypsin, other, minor, iodinated trypsin-sensitive bands were found in the fibroblast cells in the region of 180,000–200,000 (Fig. 3A).

Mesenchymal cells from liver (IAR-108, 116, 120 and 133) had iodinated protein patterns very similar to those of skin fibroblast (Fig. 3A); the only differences were in minor bands. Two lines, IAR-108 (11 weeks) and IAR-116 (2 weeks) had pronounced bands, corresponding to a component with a mol. wt of ~100,000; all other bands were in the region 160,000–170,000. Lines IAR-108 and IAR-133 also had a pronounced band, corresponding to a component with a mol. wt of 49,000 (Fig. 3A).

(ii) *Primary cultures of epithelial liver cells from adult or 5-day-old rats.*—Twelve experiments were carried out with primary epithelial liver cell cultures obtained independently from adult rats. In some experiments, the pattern of the surface proteins was investigated immediately after cell attachment (2–3 h) or after 24 h. In other experiments, each culture was iodinated at 3, 24, 48 and 72 h after cell plating, or after 5–7 days. The pattern of iodinated proteins of the adult rat liver hepatocytes in primary culture was found to be quite different from the patterns described for mesenchymal cells. The major labelled bands (Figs 3B & 4) in the gel corresponded to components with mol. wts 140,000–160,000, 100,000 and 40,000–70,000; a band that had the same position as fibronectin from mesenchymal cells was also present, but in variable and relatively small quantities. Two bands close together were seen in the region 140,000–160,000, the more intense one had a mol. wt of 152,000 and the other of 145,000. The iodinated component in the region of 100,000 was also represented as a double in some experiments at 105,000 and 98,000. Sometimes the intensities of the two latter bands were about equal, but more frequently the protein with an apparent mol. wt of 98,000 was present in greater quantities.

The pattern of iodinated proteins in the low-mol. wt region was more complex. Usually, 5–8 bands of different intensities were seen clearly in the 70,000–40,000 region. The mol. wts of the most pronounced components were: 65,000, 59,000, 54,000, 50,000 and 40,000.

The expression of these bands varied among cultures from different experiments and also during cultivation of the same cells. The doubles, 152,000–145,000 and 105,000–98,000 were the most invariable components of the various iodinated surface proteins present in these epithelial liver cells.

The $I^{125}$-labelled band corresponding in position to fibronectin was less sensitive to trypsin than fibronectin from fibroblastic cells. Other iodinated components of the hepatocytes were also less trypsin-sensitive than fibroblastic fibronectin, and could only be removed by a concentration of 50–100 $\mu$g/ml trypsin for 10 min. Sometimes this removal was incomplete, especially with the protein of mol. wt 100,000, which was never, except in one experiment, completely removed (Fig. 4). These iodinated and trypsin-sensitive components did not represent a significant portion of the total protein content of the cells, because the pattern of protein stain-

FIG. 4.—Quantitation of radioactivity of $^{125}$I-labelled bands in primary cultures of adult epithelial rat liver cells. Dried gels were cut into 1mm sections and counted in a $\gamma$-counter (●) no trypsinization after labelling; (○) 100 $\mu$g/ml trypsin for 10 min.

ing with Coomassie Blue did not change after trypsinization. Trypsin treatment after iodination (10–100 $\mu$g/ml, 37°C, 10 min) produced no new distinct bands within the hepatocyte surface-protein pattern. Treatment with thrombin (200 u/ml for 10 min) and with 1M urea (for 40 min at 37°C after iodination did not sig-

FIG. 5.—Quantitation of radioactivity of labelled bands in cultures of adult rat fibroblastic cells (IAR-109). Processed as in Fig. 4.

FIG. 6.—Quantitation of radioactivity of labelled bands in long-term cultures of epithelial rat liver cells (IAR-20). Processed as in Fig. 4.

nificantly affect the pattern of the iodinated hepatocyte proteins. Iodination of the hepatocytes after treatment with trypsin (100 μg/ml, 37°C, 10 min) or with neuraminidase (Sigma type IX, 13·3 u/ml, 22°C, 30 min) revealed no new iodinated bands, but essentially decreased the intensity of the usual bands. Iodination of cells in culture medium containing FCS instead of phosphate buffer solution did not change the pattern of iodinated proteins.

In 3 experiments with hepatocytes from 3- and 5-day-old rats (IAR-131, 138 and 140) the patterns of surface proteins were essentially the same as those of cells from adult rats.

(iii) *Long-term cultures of (nontumori-*

*genic) epithelial liver cells.*—The iodinated surface component patterns of all cell lines of this type were very similar. The total amount of iodinated surface components of the 8 untransformed liver-cell cultures examined was significantly smaller than that with primary liver-cell cultures (Fig. 6); labelled bands in the ARGs of the gels were faint. Labelled counts of these bands gave values of an order of magnitude less than analogous values for adult hepatocytes, so X-ray films were exposed for a longer period of time.

As with primary cultures of epithelial liver cells, the pattern of the iodinated proteins of the cells of the long-term cultured epithelial lines could be divided conditionally into 4 major groups of bands: a band in the region of 220,000–240,000, bands in the region of 140,000–160,000, bands with mol. wts of ~100,000, and some bands in the low-mol.-wt region (Fig. 3C). The positions of the highest mol. wt band and the band at 100,000, as well as their sensitivities to trypsin treatment, were indistinguishable from those of corresponding bands in the pattern of primary cultured hepatocytes. Cells of the epithelial lines showed a great reduction in the number and intensity of the bands in the low-mol.-wt region, where primary liver cells had the most complex pattern. Only one reproducible band, which corresponded to 50,000, was found in the low-mol.-wt region of the untransformed epithelial liver cell line patterns. The band of the same mol. wt was found in the spectra of cultures of epithelial (Fig. 3B & C) and non-epithelial (Fig. 3A) liver cells and of *in vitro* transformed epithelial cell lines (Fig. 3D).

In the region of 140,000–170,000, these long-term cultures of epithelial liver cells had 2–4 bands, but this pattern was different from the analogous pattern in primary cultures; the band in the region of 152,000 was absent or very faint, and the band in the region of 145,000 was much more pronounced. In addition, these long-term cultures of epithelial liver cells show iodinizable proteins in the region of 160,000–170,000 and a faint band around 135,000–140,000, which were practically absent from primary cultures.

The patterns of surface proteins of 3 independently obtained clonal lines (IAR-20-PC1, -PC2 and -PC3) were practically identical to the pattern of their parental line (IAR-20). Thus, it is more probable that the bands seen reflected different surface components within individual cells than the heterogeneity of the cell population.

The pattern of iodinated surface proteins was examined at different points on their growth curve in one long-term culture, namely the IAR-20 cell line. Cells were iodinated at different times, starting with the suspension just before seeding and finishing with the heavily confluent stage. A regular shift was seen from a pattern with a predominating band at mol. wt 100,000 to a pattern in which proteins with a higher mol. wt, particularly fibronectin, were prevalent. Apart from this, the total protein pattern, revealed by protein staining, remained unchanged under these experimental conditions.

No changes have been found in the surface-protein pattern of IAR-20 untransformed cell line, according to its age in culture (28–47 weeks) (Table).

(iv) In vitro *transformed epithelial liver-cell lines.*—Six transformed liver-cell lines were studied. The spectra of the iodinated proteins of the cells of this type were very similar to those of analogous untransformed cells (Fig. 3D). In one case (IAR-27 at 73 weeks in culture) some bands had disappeared in the region of 100,000–160,000 when compared with untransformed IAR-27. The cells of IAR-6-1 and IAR-2-25 had a more pronounced line at 135,000 than their untransformed prototypes. The fibronectin band was practically absent from the patterns of IAR-2-19 and IAR-2-25. The spectra of iodinated proteins of IAR-2-28 and IAR-6 (66 weeks) were nearly identical to the spectra of the parental untransformed lines (IAR-2 and IAR-6 (24–25 weeks)).

(v) *Liver tumour-cell lines.*—Ten liver tumour-cell lines were studied. Seven of them were derived from tumours produced by epithelial liver-cell lines transformed *in vitro* and 3 from liver tumours obtained *in vivo*. The patterns of the iodinated proteins of these liver tumour cells in culture were similar to those of transformed and untransformed liver-cell lines, but the bands were still less pronounced, though they maintained bands corresponding in position to fibronectin. Six such tumour-cell lines out of 7 had a major band with mol. wt ~ 100,000; these 6 also had bands with mol. wt 135,000, which were much more intense than those of untransformed lines or their parental *in vitro* transformed lines (Fig. 3E).

Two cell lines from rat liver tumours induced *in vivo* had a few iodinated proteins on the cell surface. One of these (7777) had a small amount of fibronectin and traces of iodinated material in the region of 150,000–180,000. Only a faint band was seen in the 100,000 region during electrophoresis of the 8994 tumour cells. In contrast, human hepatoma cells had pronounced iodinated bands corresponding to 100,000, 145,000 and 155,000 (Fig. 3F).

### DISCUSSION

In this paper, we have described surface-protein patterns of various normal and neoplastic rat liver-cell lines. Control experiments which were negative indicate that components of the enzyme mixture did not contribute to the cell-surface proteins patterns. Unfortunately, at present we cannot rule out the possibility that some of the surface proteins mentioned (excluding fibronectin) are absorbed serum components. Although preliminary experiments showed that all iodinated bands had biosynthetically labelled counterparts, definitive solution of this question is not possible by using whole-cell lysates containing too many different proteins of the same molecular weight. Purification of the epithelium surface proteins and re-examination of their cellular nature is now in progress.

The surface-protein pattern of mesenchymal cells from skin or liver of adult and neonatal rats (Fig. 3A) was very similar to that of other mesenchymal cells (Hynes, 1976; Vaheri & Mosher, 1978). The main enzymatically iodinated protein of these cells was found to have a mol. wt of 220,000–240,000, and was highly sensitive to trypsin. Some differences in the minor iodinated bands were seen between liver mesenchymal cells and skin fibroblasts; however, it is not possible at present to be sure whether these differences are due to specific surface proteins for both types of cell or whether they reflect heterogeneity of cell populations.

The pattern of iodinatable surface proteins of primary cultures of liver epithelial cells was quite different from and more complex than the pattern in mesenchymal cells of liver or skin. Although these cells show high-mol. wt surface proteins, with the same mol. wt as fibronectin of mesenchymal cells, the major bands were in the low-mol.-wt region of the gel (Fig. 4). A protein of 80,000 daltons was found by Marceau *et al.* (1977) to be a major band on the surface of the hepatocyte. No pronounced differences were found between the surface-protein spectra of adult and neonatal hepatocytes. Previous studies by Stenman & Vaheri (1978) showed no detectable fibronectin in the hepatocytes of liver tissue; however, Chen *et al.* (1977) described the synthesis of fibronectin in early passages of epithelial cells isolated from rat liver. Fig. 2 clearly shows the presence of fibronectin on cultured hepatocytes; the observation that epithelial liver cells have a surface protein of the same mol. wt as "fibroblastic" fibronectin, though circumstantial, is consistent with the immunofluorescence findings. In addition, surface proteins of lower mol. wt which appear to be specific for this type of epithelial cell have been found. The "epithelial" fibronectin is present in a smaller amount than the "fibroblastic" one and appears to be more resistant to trypsin.

The total amount of iodinated surface proteins in long-term cultures of epithelial liver cells is smaller than that in primary cultures. Many bands, particularly in the low-mol.-wt region of the gel, disappeared or decreased in intensity in these liver cells (Fig. 3C) but the band corresponding in position to "fibroblasts" fibronectin was retained. Although this band showed a different sensitivity to treatment with trypsin from "fibroblastic" fibronectin, it seems at present that these are not two distinct proteins. Immunofluorescent experiments with antibodies against "fibroblastic" fibronectin have revealed this protein on the surface of IAR-6 and IAR-6-1 cell lines. These cell lines maintain the typical morphology of epithelial cells after several months in culture, and when they are transformed and inoculated into syngeneic rats they give rise to well-differentiated carcinomas (Montesano *et al.*, 1977). It is not possible, however, to exclude the possibility of a specific liver surface, high-mol.-wt protein, in addition to fibroblastic fibronectin. In contrast to these observations, Chen *et al.* (1977) reported that fibronectin was lost from the surface of epithelial cells after serial passage in culture.

No major differences were observed between the surface proteins in tumorigenic and in nontumorigenic epithelial-cell lines, with the exception of an increase in the intensity of the band of mol. wt 135,000 in 2/5 transformed lines (Fig. 3D). Four out of 6 tumorigenic cell lines retained fibronectin.

The pattern of iodinated proteins of liver tumour cells in culture was also very similar to that seen in tumorigenic liver-cell lines; however, almost all the tumour cell lines contained a protein with mol. wt 135,000 as one of the major iodinated bands (Fig. 3E). Further investigations should clarify whether the increase in this surface component is linked to the tumorigenicity of the liver cells. Fibronectin, which was lost or reduced when fibroblasts were transformed *in vitro* (Hynes, 1976; Vaheri & Mosher, 1978) was retained by most *in vitro* transformed and tumour epithelial-cell lines. Recently, Wigley and Summerhayes (1979) also found no clear correlation between loss of fibronectin and the transformed phenotype in two well-characterized epithelial cell *in vitro* transformation systems.

All rat tumour-cell lines except one (IAR-2-31-RT6) had very similar, if not identical, surface-protein spectra. This indicates their common origin (epithelial), regardless of the fact that some of them (IAR-2-31-RT1 to -RT6; see Table) have a spindle-like cell morphology in culture, and that the original tumours were characterized histologically as mesenchymal or mixed tumours. The IAR-6-1-RT7 and -RT8 lines show a typical epithelial morphology in culture; and all the tumours obtained after inoculation of these cells, including those from which the cells originated, were clearly of an epithelial nature (carcinomas) (Montesano *et al.*, 1975, 1977). Although further studies on cloned cell populations from these cultures may clarify this apparent contradiction, it is also possible that the cells with a mesenchymal morphology represent a stage of undifferentiated precursors of epithelial cells.

The presence of specific liver protein markers (albumin, transferin, ligandin, A-protein and AFP) was detected only in primary cultures of liver cells from adult (with the exclusion of AFP) or 5-day-old rats, and not in tumorigenic or non-tumorigenic long-term cultures of epithelial liver cells nor in the rat liver tumour-cell cultures. Previously (Kuroki *et al.*, 1976; Huberman *et al.*, 1979) examination of several enzymes involved in glucose and amino acid metabolism in some of the long-term cultures of epithelial liver cells from adult rats had indicated that these cells acquire a foetal enzyme pattern; the activity of the foetal liver-cell enzyme, $\gamma$-glutamyl transpeptidase, was observed in a considerable proportion of transformed liver-cell epithelial cultures. In a detailed study, Sirica *et al.* (1979) reported the reversion from adult to foetal enzyme patterns in adult rat hepatocytes grown on collagen gel/nylon meshes for up to 13 days. The failure in our studies to detect specific liver-cell protein markers or AFP in long-term cultures of liver cells, could be attributed to the fact that our liver epithelial cells were not grown on a collagen gel, or were deprived of some other critical factor for the expression of these proteins.

We would like to thank Dr L. Zardi, Institute of Oncology, University of Genoa, Italy, for the anti-fibronectin antibodies; and Drs L. Tomatis and H. Yamasaki, International Agency for Research on Cancer, and J. M. Vasiliev, Cancer Research Centre, The USSR Academy of Medical Sciences, for the helpful criticism of the manuscript.

This work was supported, in part, by contract NO1-CP-55630 from the National Cancer Institute, D.H.E.W., U.S.A.

### REFERENCES

BANNIKOV, G. A. & TCHIPYSHEVA, T. A. (1972) Distribution of azo-dye binding protein in the organs of rats and mice. *Bull. Exp. Biol. Med. (U.S.S.R.)*, **77**, (In Russian).

Bannikov, G. A. & Tchipysheva, T. A. (1979) The preparation of monospecific antibodies against A-protein and immunological study on distribution of this protein in normal and tumour tissues. *Voprosy Med. Chem.*, **3**, 292.

BECKER, J. E., DE NICHAUD, G. & POTTER, V. R. (1976) Two new rat hepatoma cell lines for studying the imbalanced blocked autogeny hypothesis. In *Oncodevelopmental Gene Expression*. Eds Fishman & Sell. New York: Academic Press. p. 259.

BENENSON, A., KAPELLER, M. & DOLJONSKI, F. (1977) Surface proteins of fibroblast and sarcoma cells: Their shedding and trypsin sensibility. *Isr. J. Med. Sci.*, **13**, 852.

CHEN, L. B., MAITLAND, N., GALLIMORE, P. H. & MCDOUGALL, J. K. (1977) Detection of the large external transformation-sensitive protein on some epithelial cells. *Exp. Cell Res.*, **106**, 39.

CROUCH, E., BALIAN, G., HOLBROOK, K., DUKSIN, D. & BORNSTEIN, P. (1978) Amniotic fluid fibronectin: characterization and synthesis by cells in culture. *J. Cell Biol.*, **78**, 701.

HUBBARD, A. L. & COHN, Z. A. (1975) Externally disposed plasma membrane proteins. I. Enzymatic iodination of mouse L cells. *J. Cell Biol.*, **64**, 438.

HUBERMAN, E., MONTESANO, R., DREVON, C. & 4 others (1979) γ-Glutamyltranspeptidase and malignant transformation of cultured liver cells. *Cancer Res.*, **39**, 269.

HYNES, R. O. (1976) Cell surface proteins and malignant transformation. *Biochim. Biophys. Acta*, **458**, 73.

KESKI-OJA, J., VAHERI, A. & RUOSLAHTI, E. (1976) Fibroblast surface antigen (SF): The external glycoprotein lost in proteolytic stimulation and malignant transformation. *Int. J. Cancer*, **17**, 61.

KUROKI, T., DREVON, C., SAINT VINCENT, L., TOMATIS, L. & MONTESANO, R. (1976) Studies on the use of liver parenchymal cells in *in vitro* carcinogenesis. *Coll. Int. CRNS*, **256**, 307.

LAEMMLI, U. K. (1970) Cleavage of structural proteins during the assembly of the head of bacteriophage. *Nature*, **227**, 680.

LAISHES, B. A. & WILLIAMS, G. M. (1976*a*) Conditions affecting primary culture of functional rat hepatocytes. I. The effect of insulin. *In Vitro*, **12**, 521.

LAISHES, B. A. & WILLIAMS, G. M. (1976*b*) Conditions affecting primary cell cultures of functional adult rat hepatocytes. II. Dexamethasone-enhanced longevity and maintenance of morphology. *In Vitro*, **12**, 821.

LINDER, E., VAHERI, A., RUOSLAHTI, E. & WARTIOVAARA, J. (1975) Distribution of fibroblast surface antigen in the developing chick embryo. *J. Exp. Med.*, **142**, 41.

MARCEAU, N., ROBERT, A. & MAILHOT, D. (1977) The major surface protein of epithelial cells from newborn and adult rat livers in primary cultures. *Biochem. Biophys. Res. Commun.*, **75**, 1092.

MONTESANO, R., DREVON, C., KUROKI, T. & 5 others (1977) Test for malignant transformation of rat liver cells in culture: cytology, growth in soft agar and production of plasminogen activator. *J. Natl Cancer Inst.*, **59**, 1651.

MONTESANO, R., SAINT VINCENT, L., DREVON, C. & TOMATIS, L. (1975) Production of epithelial and mesenchymal tumours with rat liver cells transformed *in vitro*. *Int. J. Cancer*, **16**, 550.

MONTESANO, R., SAINT VINCENT, L. & TOMATIS, L. (1973) Malignant transformation *in vitro* of rat liver cells by dimethylnitrosamine and *N*-methyl-*N'*-nitro-*N*-nitrosoguanidine. *Br. J. Cancer*, **28**, 215.

QUARONI, A., ISSELBACHER, K. J. & RUOSLAHTI, E. (1978) Fibronectin synthesis by epithelial crypt cells of rat small intestine. *Proc. Natl Acad. Sci. U.S.A.*, 75, 5548.

SIRICA, A. E., RICHARDS, W., TSUKADA, Y., SATTLER, C. A. & PITOT, H. C. (1979) Fetal phenotypic expression by adult rat hepatocytes on collagen gel/nylon meshes. *Proc. Natl Acad. Sci. U.S.A.*, **76**, 283.

STENMAN, S. & VAHERI, A. (1978) Distribution of a major connective tissue protein, fibronectin, in normal human tissues. *J. Exp. Med.*, **147**, 1054.

VAHERI, A. & MOSHER, D. F. (1978) High molecular weight, cell surface-associated glycoprotein (fibronectin) lost in malignant transformation. *Biochim. Biophys. Acta*, **516**, 1.

VESTERINEN, E., LEINIKKI, P. & SAKSELA, E. (1975) Cytopathogenicity of cytomegalovirus to human ecto- and endocervical epithelial cells *in vitro*. *Acta Cytol.*, **19**, 473.

WIGLEY, C. B. & SUMMERHAYES, I. C. (1979) Loss of LETS protein is not a marker for salivary gland or bladder epithelial cell transformation. *Exp. Cell Res.*, **118**, 394.

WILLIAMS, G. M. & GUNN, J. M. (1974) Long-term cell culture of adult rat liver epithelial cells. *Exp. Cell Res.*, **89**, 139.

YAMADA, K. M. (1978) Immunological characterization of a major transformation-sensitive fibroblast cell surface glycoprotein. *J. Cell Biol.*, **78**, 520.

YAMADA, K. M. & WESTON, J. A. (1974) Isolation of a major cell surface glycoprotein from fibroblasts. *Proc. Natl Acad. Sci. U.S.A.*, **71**, 3492.

ZARDI, L., SIRI, A., CARNEMOLLA, B., COSULICH, A., VIALE, G. & SANTI, L. (1980) A simplified procedure for the preparation of antibodies to serum fibronectin. *J. Immunol. Methods*, **34**, 155.